# Dynamic Recrystallization of the Constituent γ Phase and Mechanical Properties of Ti-43Al-9V-0.2Y Alloy Sheet

**DOI:** 10.3390/ma10091089

**Published:** 2017-09-15

**Authors:** Yu Zhang, Xiaopeng Wang, Fantao Kong, Yuyong Chen

**Affiliations:** 1 State Key Laboratory of Advanced Welding and Joining, Harbin Institute of Technology, Harbin 150001, China; zhangyu88309@163.com; 2 National Key Laboratory of Science and Technology on Precision Heat Processing of Metals, Harbin Institute of Technology, Harbin 150001, China; wangxiaopeng@hit.edu.cn

**Keywords:** titanium aluminides, hot pack-rolling, dynamic recrystallization, mechanical properties

## Abstract

A crack-free Ti-43Al-9V-0.2Y alloy sheet was successfully fabricated via hot-pack rolling at 1200 °C. After hot-rolling, the β/γ lamellar microstructure of the as-forged TiAl alloy was completely converted into a homogeneous duplex microstructure with an average γ grain size of 10.5 μm. The dynamic recrystallization (DRX) of the γ phase was systematically investigated. A recrystallization fraction of 62.5% was obtained for the γ phase in the TiAl alloy sheet, when a threshold value of 0.8° was applied to the distribution of grain orientation spread (GOS) values. The high strain rate and high stress associated with hot-rolling are conducive for discontinuous dynamic recrystallization (DDRX) and continuous dynamic recrystallization (CDRX), respectively. A certain high-angle boundary (HAGB: θ = 89° ± 3°<100>), which is associated with DDRX, occurs in both the recrystallized and deformed γ grains. The twin boundaries play an important role in the DDRX of the γ phase. Additionally, the sub-structures and sub-boundaries originating from low-angle boundaries in the deformed grains also indicate that CDRX occurs. The mechanical properties of the alloy sheet were determined at both room and elevated temperatures. At 750 °C, the alloy sheet exhibited excellent elongation (53%), corresponding to a failure strength of 467 MPa.

## 1. Introduction

TiAl-based alloys with low density and excellent high-temperature properties are considered promising light high-temperature structural materials for aerospace applications, such as inlet flaps, nozzle sidewalls for turbine engines, and thermal protection systems for scramjets [1,2,3,4,5]. Several studies have focused on improving the mechanical properties of these alloys by breaking down the lamellar colonies and refining the grains via alloying-element additions [6,7], heat treatment [8,9], and thermomechanical processing (e.g., hot-forging [10], extrusion [11], rolling [12,13,14]). Hot pack-rolling processing, as the most practical hot-working method for TiAl alloy sheet production, will probably be required for the fabrication of various TiAl alloy structural components with complex shapes. However, the high tensile stress, severe heat loss, and high strain rates associated with hot-rolling, as well as the limited hot deformability of these alloys, prevent widespread use of the corresponding TiAl alloy sheets [15,16,17,18]. Recently, a novel β-γ TiAl alloy with excellent deformability has gained significant attention. This alloy is characterized by a high volume fraction of the β phase, obtained through the addition of β-stabilizing elements (such as Nb, Mo, Cr, and V [19]). The β phase, with abundant independent slip systems on the grain boundaries, plays a significant role as a lubricant during thermomechanical processing. Dynamic recrystallization (DRX) of the hard-deformed γ phase, which only has a limited number of available slip systems, is typically slower than that of the β phase. As the main mechanism for flow softening in the late stages of hot-rolling, γ-phase DRX can soften TiAl alloys during hot-rolling, resulting in grain refinement and reduced resistance to deformation [20]. Therefore, an understanding of the DRX behavior of the γ phase in the TiAl alloy sheet is essential for further optimization of the hot-rolling process parameters.

Using the ingot metallurgical (IM) method, TiAl alloy sheets are fabricated via hot isostatic pressing of the ingots and subsequent forging for pore elimination and break-down of the coarse lamellar colonies (prior to hot-rolling), respectively. These processing procedures usually lead to quite complex microstructural evolution. In addition, high-density dislocation pile-ups, shear bands, substructures, and twin boundaries are typically formed after hot-rolling [21]. The difficulty associated with investigating the effect of deformation on high-temperature microstructure is exacerbated by the complex flow behavior of TiAl alloy during hot-rolling [20]. However, the DRX behavior of the γ phase can be investigated by evaluating the hot-rolling-induced variations in the microstructure. The correlation among the hot-working parameters, microstructure, and mechanical properties of hot-deformed TiAl alloy can be simultaneously obtained. Studies based on the transmission electron microscopy (TEM) analysis method have reported that the γ phase with tetragonal L1_0_ structure has low stacking fault energy. For these alloys, DRX is generally the main dynamic softening mechanism during thermomechanical processing. In those studies, DRX during hot working was accompanied by dynamic recovery, which resulted from the inhibition of dislocation climb and provided the driving force for recrystallization [22,23]. Other studies have suggested that new grains are directly nucleated within the γ grains [24]. Although the recrystallization of TiAl alloys has been extensively investigated, the DRX behavior of the γ phase, especially in the hot-rolled TiAl alloy sheet, is only partly understood, and must therefore be elucidated.

In the present study, a vanadium-stabilized β-γ Ti-43Al-9V-0.2Y alloy sheet subjected to a total reduction of 65% was successfully fabricated via hot pack-rolling. The grain orientation spread (GOS) approach is used to distinguish between the dynamic recrystallized grains and their deformed counterparts. The microstructural evolution and DRX behavior during hot-rolling are systematically investigated, and the mechanical properties at room and elevated temperatures are determined.

## 2. Material and Experimental Procedure

**Figure 1:**
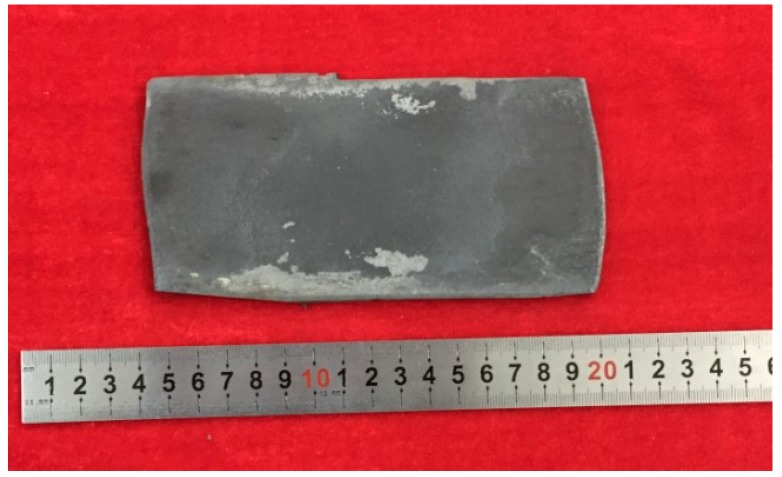
Appearance of the crack-free Ti-43Al-9V-0.2Y alloy sheet.

Ti-43Al-9V-0.2Y (at %) sheet was fabricated via hot pack-rolling after ingot casting and subsequent canned forging (details of the casting and forging processes are provided in our previous work [25]). A 75 mm × 73 mm × 10 mm rectangular billet was cut from the center of the forged TiAl pancake (Φ780 mm × 65 mm), canned by a 304 stainless steel jacket, and sealed through brazing. Afterward, the specimen was rolled at room temperature, without lubrication, on a Φ320 mm × 400 mm rolling mill. A crack-free 3.5-mm-thick TiAl sheet was obtained via eight-pass hot-rolling at 1200 °C (as shown in Figure 1), with a nominal thickness reduction per pass and rolling speed of ~15% and 50 mm/s, respectively. The specimen orientation was invariant during rolling. Prior to the initial rolling pass, the TiAl specimen was heated at 1200 °C for 1–2 h and subsequently reheated (after each rolling pass) for 10–20 min at this temperature. After the final rolling pass, the hot pack-rolled TiAl sheet was kept for 4 h at 800 °C and cooled in a furnace to eliminate the residual stress. Flat tensile specimens (gauge size: 20 mm × 5 mm × 2 mm) were cut parallel to the rolling direction (RD) of the hot-rolled sheet. These samples were subjected to tensile tests at room and elevated temperatures and initial strain rates of 1.0 × 10^−3^ s^−1^ and 5.0 × 10^−4^ s^−1^, respectively. 

The phases in the materials were identified via XRD using monochromatic Cu Kα radiation. A Quanta 200FEG scanning electron microscope (SEM) equipped with energy dispersive spectroscopy (EDS) and electron backscatter diffraction (EBSD) systems, was used to characterize the deformed microstructures, grain boundary character distributions (GBCDs), and fracture surfaces of the TiAl-alloy tensile specimens. Prior to the measurements, the SEM samples were all cut from the center of the forged pancake and hot-rolled sheet using an electro-discharge machine. Subsequently, the specimens were prepared via mechanical polishing followed by electrolytic polishing, at −20 °C and 25 V, with a solution of 10% perchloric acid +30% butanol +60% methanol. The grain size of the γ phase was measured (from SEM micrographs) via the linear-intercept method. The EBSD data were obtained from an ~150 μm × 150 μm-sized region and analyzed with TSL OIM Analysis 6.14 commercial software. The GBCD was determined based on misorientation angle (θ) classifications where, θ: 2°–15° for low-angle grain boundaries (LAGBs) and θ > 15° for high-angle grain boundaries (HAGBs). In addition, the deformation substructures and dislocations were investigated using a Tecnai G2 F30-type TEM. The TEM foils were prepared by twin-jet electro-polishing of the specimens.

The dynamic-recrystallization behavior at different rolling temperatures was analyzed based on the grain orientation spread (GOS) method, where the average difference in orientation (i.e., GOS) between the average grain orientation and all measurements in a grain is determined. The GOS is mathematically expressed as [26]

(1) $$\mathrm{GOS}=\frac{1}{N}\sum_{A=1}^{N}\left\{\min\left[\cos^{-1}\left(\frac{\mathrm{trace}\left[g_{ave}{\left(h_{i}g^{A}\right)}^{-1}\right]-1}{2}\right)\right]\right\}$$

where, *A* is the *A*-th measurement point in a grain consisting of *N* measurements, *g_ave_* is the average orientation of the grain, *g^A^* is the orientation measured at the *A*th position, and *h_i_* is the minimum misorientation angle between the average orientation and the *A*th measurement. In this approach, the GOS value of DRX grains is assumed to be lower than that of the deformed grains. The area fractions of DRX grains and deformed γ grains can both be evaluated via this approach.

## 3. Results and Discussion

### 3.1. Microstructure Evolution and Phase Composition of the Ti-43Al-9V-0.2Y Sheet

**Figure 2:**
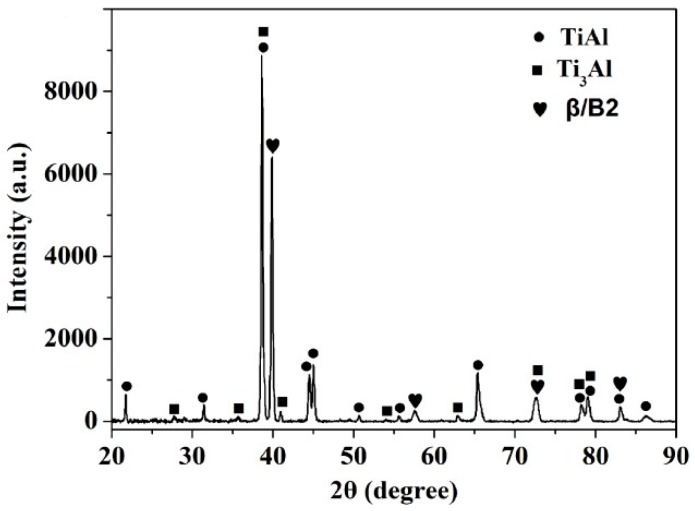
XRD pattern of the as-forged Ti-43Al-9V-0.2Y alloy.

**Figure 3:**
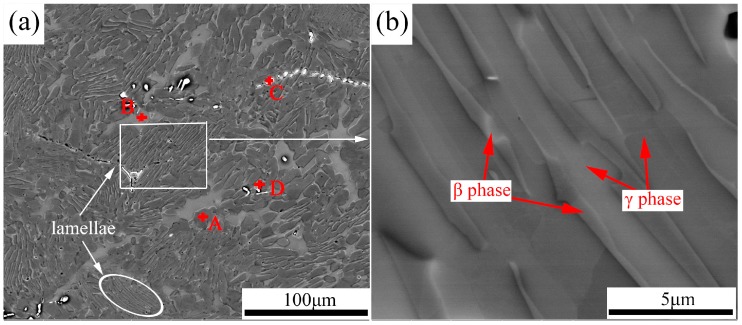
BSE images of the as-forged Ti-43Al-9V-0.2Y alloy: (**a**) low magnification; (**b**) high magnification.

The XRD pattern of the as-forged Ti-43Al-9V-0.2Y alloy (see Figure 2) reveals that the alloy is composed of γ (TiAl), β/B2, and α_2_ (Ti_3_Al) phases. The corresponding microstructures and EDS results are shown in Figure 3 and Table 1, respectively. As Figure 3 shows, the matrix consists of a β phase (gray contrast), which is distributed mainly on the γ grain boundaries, γ phase (dark contrast), and a discontinuous distribution of particles (bright contrast). Moreover, SEM examination reveals that the matrix is non-uniform, as evidenced by the high fraction of β/γ lamellar structures and heterogeneous γ grains. Figure 3b shows the residual irregular β/γ lamellar structures, which are characterized by an absence of a definite orientation relationship (OR) between the γ and β laths. The coarse and crooked β/γ morphology results from the pearlitic mode of phase transformation (α→β + γ) that occurs in the Ti-Al-M ternary system (“M” represents the β-stabilizing elements) [27]. As shown in Figure 3a, the α_2_ phase constitutes low fractions of the as-forged TiAl alloy. Furthermore, results of the EDS analysis indicate that the V content (18.5 at % in Table 1) of region B is higher than that (6.2 at %) of region A. The β phases are enriched in V, a β-stabilizing element. In addition, the bright particles, identified as Y-rich phases (probably, YAl_2_ or Y_2_O_3_) via EDS, are generated during casting, owing to the relatively low solubility of Y in the TiAl-based alloy. 

**Figure 4:**
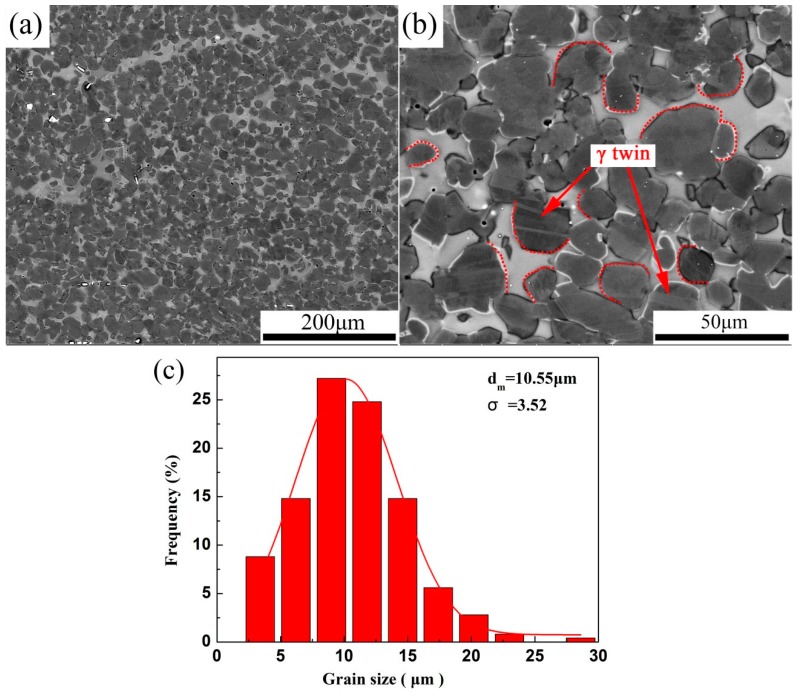
(**a**,**b**) BSE images of the hot-rolled Ti-43Al-9V-0.2Y alloy; (**c**) Area-weighted γ grain size distributions in the TiAl alloy sheet.

The microstructures resulting from hot-rolling at 1200 °C are shown in Figure 4a,b. After hot-rolling, the coarse and crooked lamellar colonies are completely broken down and a β/γ dual-phase microstructure is generated. The hot-rolled sheet has a typical recrystallized structure, where homogeneous equiaxed γ grains with some twins occur in the γ grains. An insufficiently slow rate of cooling from the hot-rolling temperature leads to incomplete diffusion-controlled phase transformation. Consequently, the massive β phase is evenly distributed at the γ-grain boundaries in the hot-rolled microstructure. The volume fraction (>20%) of the β phase is higher than that of the other β-γ TiAl alloys investigated in recent years [14,28,29]. According to previous reports, the disordered β phase provides a sufficient number of independent slip systems, and the easy dislocation climb of this phase leads to rapid dynamic recovery and DRX. The deformation structures of this phase are almost eliminated by these processes [30]. Therefore, the high-temperature soft β phase has a lower dislocation density than other phases and acts as a lubricant at the triple junctions of γ grains. Furthermore, the stress concentration on the interfaces can be relieved through the emission of dislocations from γ grains to adjacent β grains [23,31]. The relatively easy occurrence of grain boundary sliding may lead to improved deformability of TiAl alloys and prevent the formation of micro-cracks during thermomechanical processing. In addition, the β phase is metastable and the β→α_2_ and β→γ transformations are conducive for the relaxation of stresses concentrated at the phase boundaries during thermal deformation [31,32,33,34]. Figure 4b shows the γ-grain growth and β-ribbon shrinkage in the hot-rolled TiAl alloy sheet. The β→γ phase transformation may also occur during the current hot-rolling process. The equiaxed γ grain size was calculated from SEM micrographs of at least 500 grains in the hot-rolled TiAl sheet. Figure 4c shows the area-weighted γ grain size distributions (in this case, a Gaussian distribution with a single peak), average grain size (d_m_), and grain size standard deviation (σ) of the hot-rolled sheet. A d_m_ and a σ of 10.6 μm and 3.5 μm, respectively, are obtained for the globular γ grains. These values indicate that the microstructure of the TiAl alloy sheet is very fine and more homogenous than that of the as-forged alloy, which consist of coarse β/γ lamellar structures. According to previous work, a hot-rolling temperature of 1200 °C lies in the β + γ + α phase region, as revealed by differential scanning calorimetry (DSC) analysis and the ternary phase diagram proposed for the Ti-Al-V ternary system. This three-phase region can inhibit grain growth during hot-rolling [27]. In addition, the easy nucleation of DRX, owing to the high stress and strain rates associated with hot-rolling, plays a key role in the formation of the homogenous microstructure.

### 3.2. DRX of the TiAl Alloy Sheet

• EBSD Characterization of the Deformed Microstructure

**Figure 5:**
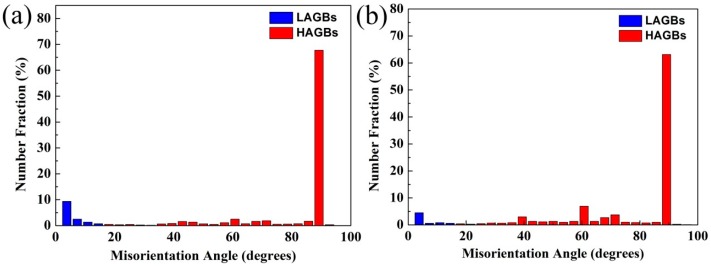
Misorientation angle distributions of the γ phase in the hot-deformed Ti-43Al-9V-0.2Y alloys: (**a**) As-forged; (**b**) Hot-rolled.

**Figure 6:**
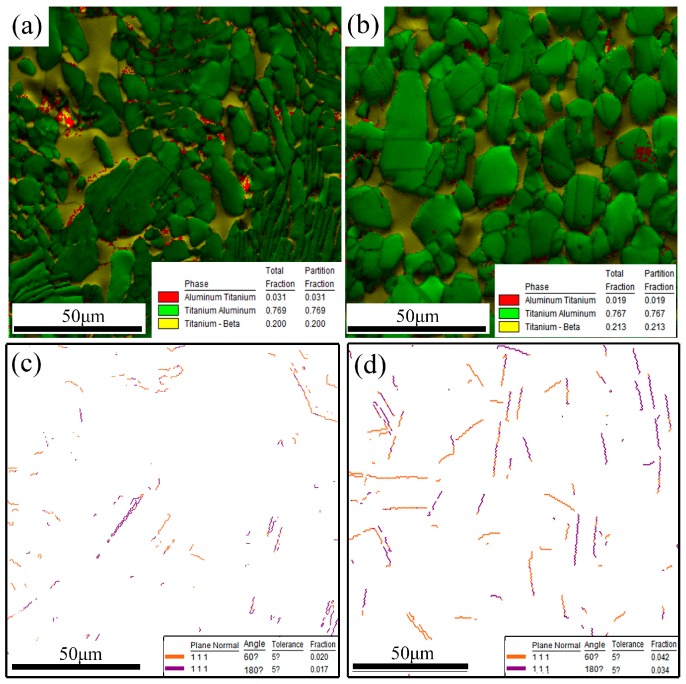
Phase composition of the Ti-43Al-9V-0.2Y alloy and twin boundary distributions of the γ phase: (**a**,**c**) As-forged; (**b**,**d**) Hot-rolled.

Figure 5 shows the misorientation angle distributions of the γ phase in the as-forged and hot-rolled TiAl alloys. Based on their misorientation angles, γ-grain boundaries are typically classified as LAGBs and HAGBs. HAGBs are mainly generated (including γ nucleus formation and the crystal growth of γ grains through swallowing and merging) via DRX. However, LAGBs are mainly characterized by the substructures generated during hot-deformation. Figure 5 shows that HAGBs constitute the main boundaries in the as-forged and hot-rolled alloys (i.e., hot-deformed materials). The LAGB fraction decreases from 9.9% to 6.2% with hot-rolling. Therefore, as indicated by the SEM micrographs, the hot-rolled TiAl alloy sheet exhibits characteristics consistent with the occurrence of γ DRX. The distribution of HAGBs consists of one prominent peak (at θ = 89° ± 3° and frequency 63.2%), which is typical of discontinuous dynamic recrystallization (DDRX) microstructures. Additionally, compared with its as-forged counterpart, the alloy sheet contains almost twice as many twin boundaries (see Figure 6). According to previous work, the twin boundaries can promote nucleation and provide ideal sites for γ grain formation during hot-working [35]. The high strain rates and high tensile stress associated with hot-rolling yield a higher volume fraction of twins in the TiAl alloy sheet than in the as-forged alloy, and are instrumental to γ DDRX. 

**Figure 7:**
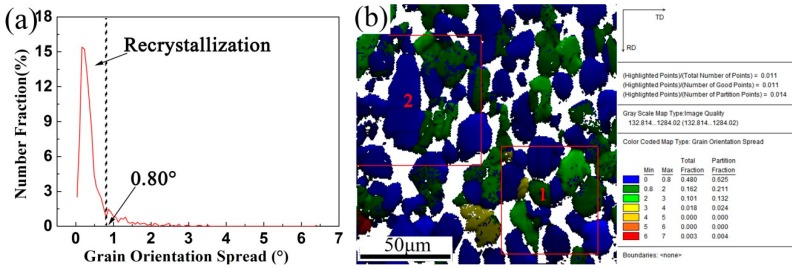
GOS distributions of the γ phase in the Ti-43Al-9V-0.2Y alloy sheet: (**a**) The curves of GOS distributions; (**b**) Maps of GOS with image quality.

Figure 7 shows the GOS distributions of the γ phase in the TiAl alloy sheet. A threshold GOS value is typically used to distinguish the recrystallized grains from their deformed counterparts. For the TiAl alloy sheet, the only prominent peak of the GOS distribution occurs at ~0.80° (Figure 7a), which can be considered a ‘cut off’ for distinguishing the DRX grains from the deformed matrix. The dynamic recrystallized grains are colored in blue (see Figure 7b). Owing to widespread DRX during hot-rolling, recrystallized γ grains constitute up to 62.5% of the microstructure. The multi-pass rolling process provides a high driving force for DRX (including both nucleation and growth) of the γ grains. 

According to previous studies [13,22], the DRX mechanism can be classified as either, continuous dynamic recrystallization (CDRX) or DDRX. In general, CDRX is closely correlated with slow dynamic recovery (DRV), which occurs through continuous absorption of dislocations in the substructures (LAGBs). This process (i.e., DRV) can provide sufficient driving force for the continuous formation of new recrystallized grains, whereas DDRX is characterized mainly by nucleation and grain growth processes that proceed through the sweeping action of HAGBs.

**Figure 8:**
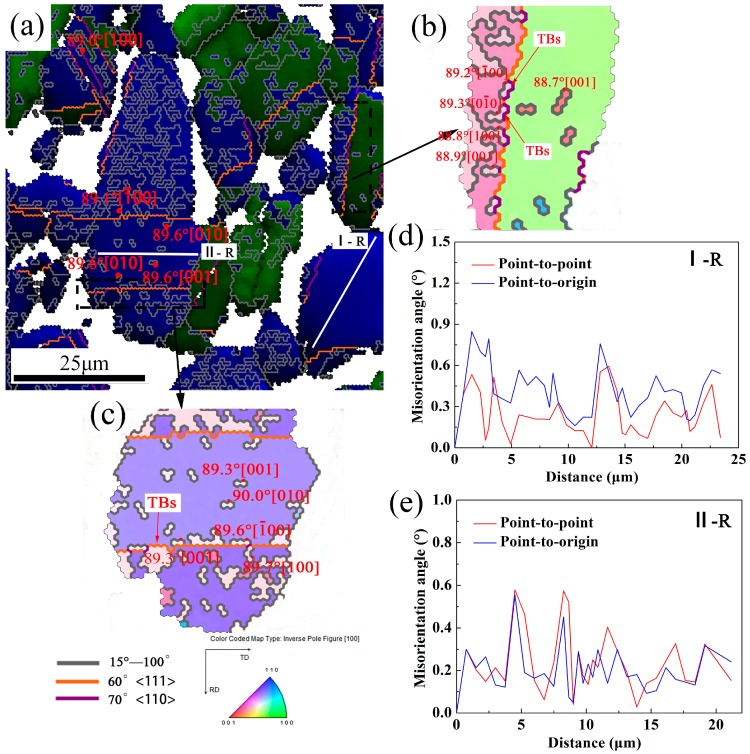
(**a**) A magnified view of the region 2 (see Figure 7) with HAGBs (HAGBs: gray lines; twin boundaries: orange and purple lines); (**b**,**c**) The inverse pole figures corresponding to a single deformed grain and a recrystallized grain, respectively (twin boundaries: TBs); (**d**,**e**) Misorientation measured along the lines denoted as I-R and II-R in the recrystallized grains (recrystallized grain “R”).

As previously stated, the highest peak in the grain boundary distribution corresponding to the γ phase of the TiAl alloy sheet occurs at a misorientation angle of θ = 89° ± 3°. Figure 8a shows the HAGBs and twin boundaries (indicated by gray and orange lines, respectively) in the γ phase. Twins occur in both the recrystallized grains and the deformed grains. The twins in the γ phase of the TiAl alloy result mainly from DRX grain growth and HAGB migration during DRV [24]. Figure 8d,e show the misorientation measured along the lines denoted as I-R and II-R in the recrystallized grains (GOS: <0.8°). A maximum misorientation angle of <2° reveals that substructures are absent from the interior of I-R and II-R grains. Furthermore, boundaries described by 89° ± 3°<100> misorientations occur predominantly in dynamically recrystallized grains located in the matrix grains. The sudden change in the misorientation angle of the HAGBs, via nucleation and nucleus growth in the clean DRX grains, indicates that the γ phase undergoes DDRX during hot-rolling. This is consistent with the results reported in previous studies (Zong et al. [24]). Figure 8b,c show the inverse pole figures corresponding to a single deformed grain and a recrystallized grain, respectively. As the figure shows, the large grains are divided (by HAGBs and twin boundaries) into several crystallographically oriented parts and DDRXed grains nucleate with a misorientation angle of 89° ± 3°<100> and subsequently grow. The twin boundaries also induce the nucleation of new DDRX γ grains (see Figure 8b,c). These twin boundaries provide preferential nucleation sites for DRX, as reported for magnesium alloys [36,37]. In the current alloy, these boundaries are conducive for DDRX nucleation in the γ phase during hot-rolling. 

**Figure 9:**
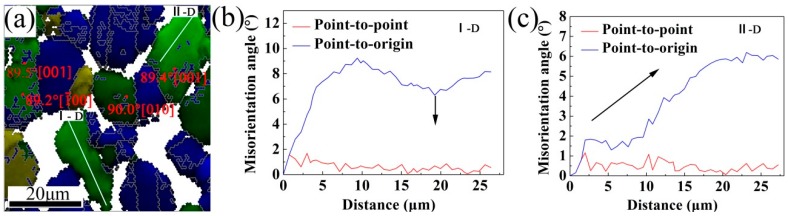
(**a**) A magnified view of region 1 (see Figure 7) with HAGBs (HAGBs: gray lines); (**b**,**c**) Misorientations measured along the lines denoted as I-D and II-D within the deformed grains (deformed grain “D”).

Figure 9 shows a magnified view of the region denoted as 1 in Figure 7b, with grain boundaries corresponding to θ > 15°. The occurrence of several new nucleated grains in deformed grains with HAGBs of θ = 89° ± 3°<100> (see Figure 9a) indicates that DDRX also occurs in the deformed grains. For the γ phase, the activation of CDRX during hot-rolling is also expected. CDRX is considered a DRV-dominant process, where the misorientation values of LAGBs increase progressively and new geometrically necessary boundaries (GNBs) are simultaneously formed [38]. In this study, substructure development within the deformed grains of the hot-rolled TiAl alloy sheet is analyzed further via point-to-point and point-to-origin misorientations. Figure 9b,c show the misorientations measured along the lines denoted as I-D and II-D within the deformed grains (GOS: >0.8°). Two types of deformation patterns are identified, namely: (i) point-to-point misorientations lower than 2° (see Figure 9b) and point-to-origin misorientations (θ: 2–15°) that increase discontinuously with increasing distance. The point-to-origin data indicate that separate regions with negligible gradients are formed within the deformed grains and intragranular sub-boundary formation leads to a fragmented-grain appearance; (ii) Point-to-point misorientation lower than 2°, but the point-to-origin misorientation increases continuously with increasing distance (see Figure 9c). This indicates that GNBs are formed in the deformed grains and accommodate the plastic strain between neighboring points [38,39]. The high strain rate and high tensile stress associated with the hot-rolling process provide sufficient drive force for the transformation of LAGBs into HAGBs. Once HAGBs are generated, new recrystallized grains form and subsequently grow through substructure absorption from the deformed grains [39]. In general, grain boundaries, phase boundaries, and high-density dislocation regions constitute excellent nucleation sites.

• TEM Characterization of the Deformed Microstructure

**Figure 10:**
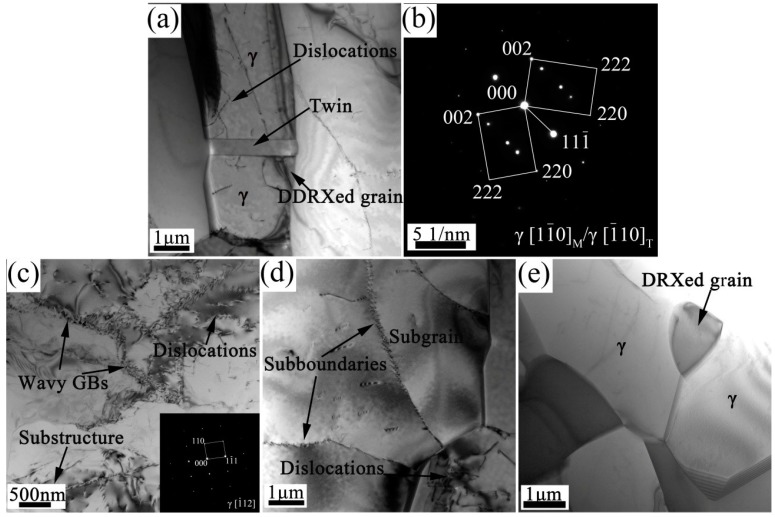
TEM images of the Ti-43Al-9V-0.2Y alloy sheet: (**a**,**b**) γ twins and corresponding SAD pattern; (**c**) High-density dislocations and substructures within the γ grain and SAD pattern of the γ phase; (**d**) γ sub-grains; (**e**) The recrystallized γ grains.

Figure 10 shows TEM images of the deformed microstructure and the corresponding selected area diffraction (SAD) pattern. Figure 10a,b show the γ twins, which are formed during hot-rolling. New γ grains are generated at the twin boundaries, which induce DDRX nucleation in the γ phase (as suggested by the EBSD results presented in Figure 8). These boundaries can provide a high driving force for the nucleation and growth of DDRX grains. Compared with the as-forged TiAl alloy, the hot-rolled sheet with a higher fraction of twin boundaries has a more completely recrystallized microstructure and, consequently, a higher number of HAGBs (see Figure 5). 

In addition, high-density dislocations and substructures (separated by wavy grain boundaries in some cases) occur within the γ grains after hot-rolling, as shown in Figure 10c. These results suggest that the multi-pass rolling process can further enhance dislocation glide and climb, as well as increase the DRV rate (in preparation for the CDRX process). The HAGB grain is separated into several parts by straight and clear sub-boundaries (LAGBs), as shown in Figure 10d, and sub-grains are generated within the γ grains. The occurrence of intragranular GNBs is consistent with the EBSD results (see Figure 9), where point-to-origin misorientations of 2–15° were obtained. Through substructure absorption near the grain boundaries, the LAGBs transform into HAGBs, leading to the formation and growth of new grains. These defect-free recrystallized grains nucleate at the triangular grain–boundary interfaces (Figure 10e). This is attributed to the accommodation of severe plastic deformation during hot-rolling, where dislocation motion and DRX behavior are required for the release of stress concentration and softening of the metal matrix, respectively. This, in turn, prevents crack formation in the TiAl sheet.

The CDRX process results from the high stress generated during thermal deformation and proceeds via continuous absorption of dislocations from the grain boundaries [22]. When the dislocation density exceeds the absorption capacity of the boundaries, the rearrangement of piled-up dislocations leads to the formation of low-angle substructures, as depicted in Figure 10c,d. Subsequently, sub-boundaries transform into HAGBs of the recrystallized grains (see Figure 10e), thereby releasing the strain energy and reducing the stress concentration. Grain boundaries or sub-boundaries usually act as preferential sites for DRX nucleation. In addition, equiaxed γ grains resulting from uniform DRX of the TiAl alloy sheet can further promote grain-boundary movement and grain rotation, thereby improving the degree of DRX. In the current study, DRV in the γ phase occurs during hot-pack rolling, but proceeds very slowly as inhibition of climb and cross-slip yields a constant driving force for the CDRX process [40].

### 3.3. Mechanical Properties of the Ti-43Al-9V-0.2Y Sheet

**Figure 11:**
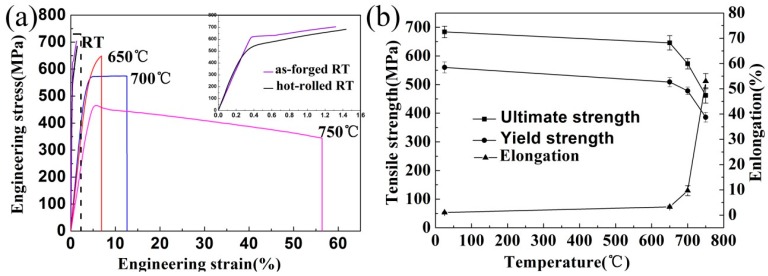
Tensile properties of the TiAl alloy: (**a**) tensile curves of the hot-deformed TiAl alloys; (**b**) the tensile-property variation of TiAl alloy sheet with increasing temperature.

The tensile properties of the deformed Ti-43Al-9V-0.2Y alloy were determined at room and elevated temperatures and strain rates of 1 × 10^−3^ s^−1^ and 5.0 × 10^−4^ s^−1^, respectively. The tensile stress–strain curves of samples tested at various temperatures are shown in Figure 11. Values of 684 MPa, 563 MPa, and 1.02% were obtained for the ultimate tensile strength (UTS), yield strength (YS), and elongation, respectively, of the hot-rolled TiAl alloy at room temperature. This elongation is slightly higher than that (0.87%) of the as-forged alloy. After hot-rolling, the homogeneous microstructure consisting of fine γ grains (especially the equiaxed recrystallized γ grains) and net-like distributed β grains contributes primarily to improved elongation of the TiAl alloy sheet. 

The tensile strength of the TiAl alloy sheet decreases with increasing temperature, whereas the elongation increases continuously (Figure 11a,b). At elevated temperatures, the hot-rolled sheet exhibits excellent ductility (for example, at 750 °C, failure strength: 467 MPa, elongation: 53%, which is higher than that of other recently investigated TiAl alloys as shown in Table 2) [20,28,41]. This excellent high-temperature elongation may be attributed to the uniform microstructure of the sheet, which is conducive for the reduction of stress concentrations, thereby delaying crack formation and propagation during tensile testing at elevated temperatures. Furthermore, the uniform equiaxed γ grains resulting from DRX are easily rotated during testing and are helpful in reducing the dislocation pile-ups.

**Figure 12:**
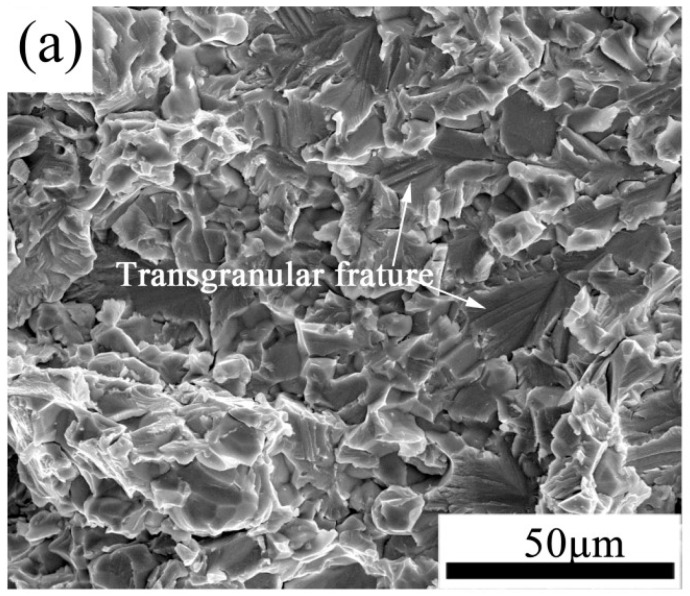

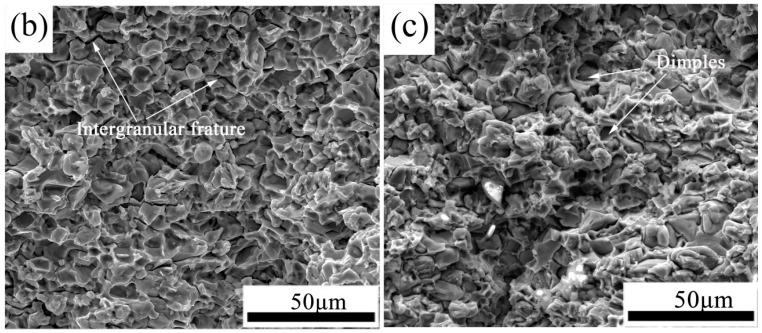
Fracture surfaces of the tensile-test samples at different temperatures: (**a**) At room temperature; (**b**) 700 °C; (**c**) 750 °C.

The fracture surfaces of the tensile-test samples are shown in Figure 12. As Figure 12a shows, the surface of the hot-rolled alloy sheet underwent mainly transgranular fracture at room temperature. This indicates that crack propagation in the sheet proceeds through a mixture of transgranular and intergranular fracture modes, owing to the duplex microstructure resulting from hot-rolling in the β + γ + α phase region. When the temperature is increased to 700 °C, the sheet undergoes intergranular fracture and exhibits an elongation of 9.7%, indicating that the hot-rolled sheet undergoes brittle fracture at temperatures below 700 °C. However, at 750 °C (see Figure 12c), many dimples form on the surface of the sheet cross-section and a significantly higher elongation (53%), than that occurring at 700 °C, is achieved. This indicates that the fracture mode of the TiAl alloy sheet has undergone a brittle to ductile transition, and the dislocation slip is the main deformation mechanism when the temperature is above 750 °C. The recrystallization nucleation, which can effectively relieve the stress concentration, occurs owing to the driving force provided by the high density dislocation pile-ups. Besides, the relatively easy rotation of the fine equiaxed γ grains in the alloy sheet also contributes to the improved tensile ductility at 750 °C. The brittle ductile transition temperature of Ti-43Al-9V-0.2Y alloy sheet lies between 700 °C and 750 °C. 

## 4. Conclusions

The DRX behavior of the constituent γ phase and mechanical properties of a hot-rolled Ti-43Al-9V-0.2Y alloy sheet are systematically investigated. The main conclusions of the study are summarized as follows:

1. The volume fraction of dynamically recrystallized grains increases after hot-rolling and multi-pass rolling promotes DDRX of the γ phase during hot-rolling. Furthermore, the θ = 89° ± 3°<100> misorientation angle occurs in both the DRX grains and the deformed grains. The twin boundaries are conducive for the occurrence of DDRX and provide ideal nucleation sites for γ grains.
2. The dislocations, sub-grain boundaries, and GNBs within the deformed grains are generated by the high strain rates and high stress associated with the hot-rolling process. These strain rates and stress provide a continuous driving force for the transformation of LAGBs into HAGBs, in preparation for the CDRX process. The DRX of the γ phase in the current TiAl alloy sheet results from the occurrence of both DDRX and CDRX during hot-rolling and plays a key role in microstructure refinement.
3. The Ti-43Al-9V-0.2Y alloy sheet exhibits a UTS and ductility of 684 MPa and 1.02%, respectively, at room temperature. When the tensile-test temperature is increased to 750 °C, the sheet exhibits an excellent elongation (53%), with a failure strength of 467 MPa. The brittle–ductile transition temperature of the TiAl alloy sheet lies between 700 °C and 750 °C.

## Figures and Tables

**Table 1:** EDS analyses of grains, lamellae, and particles in the as-forged Ti-43Al-9V-0.2Y alloy.

Point	Alloying Element (at %)				
	Al	Ti	V	O	Y
**A**	45.8	48.0	6.2	-	-
**B**	32.6	49.0	18.5	-	-
**C**	60.5	10.3	2.3	-	26.9
**D**	0.7	1.2	-	58.5	39.7

**Table 2:** Microstructure and tensile properties compared with other TiAl alloys.

Alloy	Microstructure	750 °C			Reference
		YS (Mpa)	UTS (Mpa)	δ (%)	
**Ti-45Al-7Nb-0.3W**	Duplex	570	760	8	[20]
**Ti-43Al-2Cr-2Mn-0.2Y**	Nearly lamellar	-	410	43	[28]
**Ti-44Al-8Nb-(W,B,Y)**	Nearly lamellar	-	980	13	[41]
**Ti-43Al-9V-0.2Y**	Duplex	419	467	53	Current alloy
